# Effects of supplementing hemp (*Cannabis sativa* L.) leaves in concentrate diet on carcass traits and meat characteristics of *Longissimus* muscle in goat kids

**DOI:** 10.1371/journal.pone.0337125

**Published:** 2025-12-11

**Authors:** Chanporn Chaosap, Katatikarn Sahatsanon, Kazeem D. Adeyemi, Korawan Sringarm, Jamlong Mitchaothai, Achara Lukkananukool

**Affiliations:** 1 Department of Agricultural Education, School of Industrial Education and Technology, King Mongkut’s Institute of Technology Ladkrabang, Bangkok, Thailand; 2 Department of Animal Production, Faculty of Agriculture, University of Ilorin, Ilorin, Kwara State, Nigeria; 3 Department of Animal and Aquatic Sciences, Faculty of Agriculture, Chiang Mai University, Chiang Mai, Thailand; 4 Department of Animal Technology and Fishery, School of Agricultural Technology, King Mongkut’s Institute of Technology Ladkrabang, Bangkok, Thailand; Universitas Sebelas Maret, INDONESIA

## Abstract

The influence of dietary hemp (*Cannabis sativa* L.) leaf (HL) supplementation in a concentrate diet on carcass traits, physicochemical properties, amino acids, ribonucleotides, lipid oxidation, and fatty acids of *Longissimus thoracis et lumborum* (LTL) muscle in goat kids was evaluated. Fifteen entire Thai native x Boer bucks (body weight: 16.28 ± 0.35 kg; age: 4 months) were randomly assigned to concentrate diets containing 0% (HL-0), 2% (HL-2), or 4% (HL-4) HL for 150 days, after which they were slaughtered. The LTL muscles were assessed for physicochemical properties, fatty acids, amino acids, ribonucleotides and lipid oxidation. The HL-4 goats had heavier hot and cold carcass weights compared to the other groups (*P* < 0.05). Shoulder percentage was lower in the HL-0 goats than in the other groups (*P* < 0.05). The diets did not affect color, chemical composition, shear force, muscle fiber diameter, sarcomere length, ribonucleotides, or free and total amino acids in the LTL muscle in goats. However, the HL-4 meat had higher (*P* < 0.05) pH 24 h compared to the HL-2 meat, while the pH of the HL-0 meat did not differ from that of the other groups. The HL-4 meat had lower cook loss compared to the other meats (*P* < 0.05). HL supplementation increased C20:4n-6 concentration (*P* < 0.05). HL-2 meat had higher total polyunsaturated fatty acids compared to the other groups (P < 0.05). The HL-2 and HL-4 meats had lower TBARS values (*P* < 0.05) throughout the 5-day *postmortem* chill storage. Dietary inclusion of 2–4% hemp leaves improved carcass yield, fatty acids, and oxidative stability of goat meat without compromising other quality traits, suggesting its potential as a natural feed additive to enhance meat quality and shelf life.

## 1. Introduction

The livestock industry is constantly seeking innovative ways to enhance animal production and meat quality while reducing feed costs and utilizing agricultural by-products sustainably [1,2]. One such opportunity arises from the cultivation of hemp (*Cannabis sativa* L.), which has recently gained significant momentum in Thailand. In recent years, hemp has garnered increasing interest in agriculture due to its versatile applications and favorable environmental impact [3]. The Thai government is actively promoting and supporting the cultivation of hemp as an economic crop due to its wide-ranging applications, including fiber, seed, oil, and medicinal use [4]. The increased cultivation has led to a surplus of hemp by-products, such as leaves, which are rich in bioactive compounds and nutrients [5]. These by-products present a potential feed resource for livestock, which could reduce waste and offer additional nutritional or functional benefits to the animals [6,7].

Hemp by-products are rich in bioactive compounds, particularly polyphenols and flavonoids [8–11]; however, while various parts of the hemp plant have been studied in livestock nutrition [12–15], the effects of hemp leaves on carcass traits and meat quality in goats remain underexplored. Goats are highly adaptable livestock, providing crucial economic and food security benefits, particularly in arid and resource-poor regions [16,17]. Their meat, known for its lean quality and rich flavor, is a nutritious source of protein, low in fat and cholesterol compared to other red meats [16,18]. With the growing demand for sustainable and healthy meat options, goat meat has gained prominence in global markets, contributing to diversified diets and sustainable farming practices [18,19]. The inclusion of hemp leaves in goat diets presents a promising avenue for enhancing both carcass and meat quality. The unique composition of hemp leaves, particularly its phytochemical profile and antioxidant properties [8–11], could improve meat quality and oxidative stability while potentially reducing the need for synthetic additives. This approach aligns with the growing consumer demand for natural, sustainable, and high-quality meat products, and it holds particular relevance in regions like Thailand, where hemp cultivation is being promoted. Exploring the feasibility of hemp leaf supplementation in goat diets could thus offer both economic and environmental advantages, contributing to more sustainable livestock production systems.

Although the influence of dietary supplementation with various parts of the hemp plant on carcass and meat quality of small ruminants has been documented [12–15], the use of hemp leaves remains limited [20,21]. Notably, dietary inclusion of 7.4% hemp leaves (Santhica 27 variety) in dairy cows enhanced antioxidant capacity and increased the polyunsaturated fatty acid content of milk [22]. Building on this evidence, the inclusion levels of 2% and 4% hemp leaves were selected for this study to ensure a meaningful supply of phytochemicals while maintaining the isocaloric and isonitrogenous composition of the basal diets. These inclusion levels also comply with regulatory guidelines by keeping the Δ⁹-tetrahydrocannabinol (Δ⁹-THC) concentration below the 10 mg/kg (0.001%) threshold recommended for livestock feed [23,24]. In Thailand, the Department of Livestock Development permits the use of certain hemp-derived materials in animal feeds provided that the hemp product itself contains no more than 0.2% Δ⁹-THC by weight [25]. Based on the nutritional and phytochemical composition of hemp leaves and their antioxidant potential, we hypothesized that dietary supplementation with hemp leaves would enhance carcass traits, and improve meat quality and oxidative stability in goats. The objectives of this study were to examine the influence of supplementing hemp leaves in concentrate diets on carcass traits and meat characteristics of *Longissimus* muscle in goats.

## 2. Materials and methods

### 2.1. Hemp leaf collection, phytochemical and antioxidant analysis

The hemp leaves (Charlotte Angel variety) used in this study were provided by Amber Farm Co., Ltd., Thailand. The leaves were cool-dried at 20°C and 55% relative humidity for 7 days, then ground into a fine powder using a hammer mill (Retsch SM100, Retsch GmbH, Haan, Germany) equipped with a 2-mm sieve. The resulting hemp leaf (HL) powder was stored at room temperature in airtight bags until further analysis. For each feed sample, 10 g were used for phytochemical analysis and another 10 g for cannabinoid analysis, with each analysis conducted in triplicate.

Total phenolic content was determined according to the method of Norkeaw et al. [26]. Briefly, 12.5 µL of an aqueous sample was mixed with 501 µL of distilled water and 12.5 µL of Folin–Ciocalteu reagent. After 6 min, 125 µL of 10% sodium carbonate (w/v) and 100 µL of distilled water were added. The mixture was incubated at room temperature for 90 min, and absorbance was read at 750 nm. Results were calculated using a gallic acid calibration curve and expressed as millimoles of gallic acid equivalents per gram (mM GAE/g) of dried sample.

Antioxidant capacity was assessed using three assays, as described by Norkeaw et al. [26] and Wisetkomolmat et al. [27]. First, the 2,2-diphenyl-1-picrylhydrazyl (DPPH) assay was performed by mixing 50 µL of ethanolic extract with 200 µL of 0.6 mM DPPH solution and incubating the mixture in the dark at 25°C for 30 min. Absorbance was measured at 517 nm. Ethanol and DPPH without extract served as the control. Results were expressed as millimoles of Trolox equivalents per gram (mM TE/g) of dried sample. Second, the 2,2-azinobis-3-ethylbenzothiazoline-6-sulfonic acid (ABTS) assay, the ABTS stock solution was prepared by mixing 7.4 mM ABTS and 2.6 mM potassium persulfate, incubated in the dark for 12 h. The ABTS working solution was prepared by diluting 1 mL of the stock solution with 65 mL of methanol to obtain an absorbance of 0.7 ± 0.02 units at 734 nm. Then, 10 µL of methanolic extract was combined with 190 µL of ABTS working solution, incubated at 25°C for 10 min in the dark, and absorbance was measured at 734 nm. Results were expressed as mM TE/g. Third, the ferric reducing antioxidant power (FRAP) assay involved mixing 2.85 mL of FRAP working solution (acetate buffer, ferric tripyridyltriazine, and FeCl₃ in a 10:1:1 ratio) with 150 µL of the sample extract. After a 4-minute incubation at 25°C, absorbance was measured at 593 nm. Results were reported as millimoles of Fe² ⁺ equivalents per gram (mM Fe² ⁺ /g) of dried sample.

Cannabidiols contents (Cannabidiolic acid (CBDA), Cannabidiol (CBD), Cannabinol (CBN), Δ⁹-tetrahydrocannabinol acid (THCA)) and Δ⁹-THC were quantified using high-performance liquid chromatography with UV detection (HPLC-UV; Shimadzu LC-20A, Japan), following the method described by Sopian et al. [28]. One gram of HL powder was extracted with 100 mL of 80% methanol, shaken at 60°C for 1 h, filtered, and diluted to 100 mL with 80% ethanol. After filtration through a 0.45 µm membrane, 5 µL of the extract was injected into the HPLC system. Separation was achieved using a Raptor ARC-18 column (150 × 4.6 mm, 2.7 µm; Restek, USA) maintained at 30°C, with an isocratic mobile phase consisting of 25% water (containing 5 mM ammonium formate and 0.1% formic acid) and 75% acetonitrile (with 0.1% formic acid), at a flow rate of 1 mL/min. Detection was carried out at 228 nm.

For quantification, analytical-grade standards of CBD (≥99% purity) and Δ⁹-THC (≥98% purity) (Sigma-Aldrich, St. Louis, MO, USA) were used to construct calibration curves. Calibration standards were prepared in 80% methanol across six concentrations (0–50 μg/mL) and analyzed in triplicate. Analyte identification was based on matching retention times with those of the standards and confirmed by spike recovery. The relationship between peak area and concentration was linear for all analytes, with correlation coefficients (R²) as follows: CBD (R² > 0.998), Δ⁹-THC (R² > 0.998), CBDA (R² = 0.999), CBN (R² = 1), and THCA (R² = 0.999) over their respective calibration ranges. The limits of detection (LOD) and quantification (LOQ) were as follows: CBD (LOD = 0.68 μg/mL, LOQ = 2.06 μg/mL), Δ⁹-THC (LOD = 1.14 μg/mL, LOQ = 3.46 μg/mL), CBDA (LOD = 2.64 μg/mL, LOQ = 7.99 μg/mL), CBN (LOD = 0.91 μg/mL, LOQ = 2.77 μg/mL), and THCA (LOD = 2.12 μg/mL, LOQ = 6.42 μg/mL).

### 2.2. Ethics approval and consent to participate

This study was approved by the Animal Care and Use Committee, King Mongkut’s Institute of Technology Ladkrabang (ACUC-KMITL-RES/2022/017). All procedures complied with institutional and national guidelines for the care and use of animals in research. No owner consent was required because the animals were owned by the university research farm.

### 2.3. Goats and management

Fifteen Thai native × Boer crossbred male goat kids (initial BW: 16.28 ± 0.35 kg; age: 4 months), with five goat kids assigned to each dietary treatment, were used in this study. The goat kids were housed individually in pens equipped with drinking and feeding facilities. The concentrate diet, formulated according to NRC [29] guidelines, was prepared using a horizontal double ribbon mixer and provided in mash form. Goat kids were randomly assigned to one of three dietary treatments: 0% (HL-0), 2% (HL-2), or 4% (HL-4) hemp leaves in the concentrate. Each goat received concentrate at 1% of its body weight and had *ad libitum* access to corn silage for 150 days. The concentrate was fed in two equal portions at 7:30 AM and 4:00 PM. All goats had continuous access to clean water and a mineral block. The chemical composition of the diets was analyzed following AOAC [30] procedures and is presented in Table 1, while the fatty acid profiles are shown in Table 2.

**Table 1:** Ingredients and chemical composition of dietary treatments, hemp leaves, and silage.

Item	Level of hemp leaves (%)			Hemp leaf	Silage
	0	2	4		
Ingredients					
Cassava chip	35.00	35.00	35.00		
Corn meal	15.00	15.00	15.00		
Rice bran	18.00	18.00	18.00		
Palm meal	15.00	14.00	13.00		
Soybean meal	15.00	14.00	13.00		
Hemp leaf	0.00	2.00	4.00		
Molasses	1.00	1.00	1.00		
Urea	1.00	1.00	1.00		
Chemical composition (%)					
Dry matter	88.12	88.73	89.17	89.39	24.72
Crude protein	16.09	15.84	16.01	29.28	8.55
Nitrogen free extract	55.70	55.28	54.21	30.53	40.76
Ether extract	5.32	5.59	5.52	3.40	1.91
Neutral detergent fiber	23.52	23.67	23.60	18.90	57.64
Acid detergent fiber	10.27	11.10	11.42	11.01	32.50
Acid detergent lignin	2.49	2.64	2.71	1.86	3.97
Ash	5.16	6.05	6.84	17.70	7.32
Gross energy (cal/g)	3908.2	3862.9	3864.5	3600.20	

**Table 2:** Fatty acids, antioxidants and cannabinoid composition of dietary treatments and hemp leaf.

Fatty acid (%)	Level of hemp leaves (%)			Hemp leaf
	0	2	4	
C14:0	1.74	1.65	1.75	0.04
C15:0	0.32	0.29	0.31	0.00
C16:0	20.02	19.72	20.11	8.00
C16:1	2.15	2.01	2.40	0.09
C17:0	0.45	0.43	0.46	0.04
C17:1	0.00	0.00	0.00	0.03
C18:0	5.33	5.78	5.94	1.62
C18:1n9c	26.58	26.52	25.83	16.46
C18:2n6c	36.16	35.24	35.28	13.08
C18:3n6	1.59	1.5	1.65	0.75
C18:3n3	2.25	2.16	2.27	58.75
C20:1n9	0.46	0.49	0.46	0.41
C20:2	0.45	0.53	0.60	0.21
C20:3n6	0.63	0.60	0.70	0.00
C21:0	0.05	0.06	0.06	0.15
C22:0	0.00	0.00	0.00	0.39
C23:0	0.57	0.64	0.68	0.00
C24:0	0.24	1.31	0.25	0.13
Antioxidants				
Total polyphenol (mM GAE/g sample)	1309	1404	1622	4821
ABTS (IC50, mg/ml)	35.40	33.70	32.10	5.50
DPPH (IC50, mg/ml)	32.40	38.70	22.20	15.10
FRAP (mM Fe^2 + ^/g sample)	407.79	1253.75	1727.56	1208.26
Cannabinoids (mg/100 g)				
Cannabidiolic acid (CBDA)	0.00	0.10	0.14	1.88
Cannabidiol (CBD)	0.00	1.47	3.63	14.05
Cannabinol (CBN)	0.00	0.10	0.16	5.63
Δ^9^-tetrahydrocannabinol (9-THC)	0.00	0.36	0.55	13.89
Δ^9^-tetrahydrocannabinolic acid A (THCA)	0.00	0.15	0.28	9.37

DPPH, 2,2-diphenyl-1-picrylhydrazyl. FRAP, ferric reducing antioxidant power. ABTS, 2,2′-azinobis-3-ethyl-benzothiazoline-6-sulfonic acid.

### 2.4. Slaughtering, Muscle collection and Sample preparation

On day 150, goats were fasted for 12 h but had unrestricted access to water and slaughtered following the Halal procedure. After slaughter, carcasses were split and refrigerated (1-4^o^C) overnight. The *Longissimus thoracis et lumborum* (LTL) muscle was collected from the carcass to assess meat characteristics. Each left LTL muscle was divided into three 3-cm-thick slices. Instrumental color, pH, Warner-Bratzler shear force (WBSF) and cook loss were measured from two of the 3-cm-thick slices. The third slice was for measuring muscle fiber diameter and sarcomere length. The LTL muscle from the right carcass was divided into one 5-cm-thick slice for TBARS analysis and one 3-cm-thick slice for fatty acid composition, ribonucleotide content, and total and free amino acids.

### 2.5. Meat quality analyses

Muscle pH at 45 min and 24 h postmortem was measured using a pH meter equipped with a spear-tip glass electrode (SevenGo, Mettler-Toledo International Inc., Greifensee, Switzerland) with automatic temperature compensation. Before measurement, the pH meter was calibrated using standard buffer solutions with pH values of 4.01 and 7.01 at 25°C [31]. The CIE L*, a*, and b* color coordinates was measured with a spectrophotometer (MiniScan EZ, D65 illuminant, 10° observer, Hunter Associates Laboratory Inc., Reston, USA) with a 2.54 cm diameter aperture [31]. Triplicate readings were taken from different points on a sample after 45 min blooming at 25°C.

For cooking loss, only one batch was used, and the endpoint temperature was measured for each sample. A 100 g sample was put in a polyethylene bag, sealed, and cooked in a water bath at 80°C until the core temperature reached 70°C. Cooking loss was determined by calculating the percentage difference between the pre-cooked and cooked sample weights [32]. For shear force, each cooked sample was sectioned into eight cubes (1.3 x 1.3 x 3 cm), oriented perpendicular to the muscle fibers. Each cube was sheared with a texture analyzer (EZ-SX, Shimadzu, Kyoto, Japan) equipped with a 50 kg load cell and operated at a crosshead speed of 50 mm/min [32].

### 2.6. Muscle fiber diameter and sarcomere length analyses

Sarcomere length was measured by the helium-neon laser diffraction technique [33] with measurements based on an average of 10 sarcomere lengths per sample. For muscle fiber diameter, samples were fixed in 10% formalin for 48 h and subsequently blended with 0.9% sodium chloride using a laboratory blender. Diameters of 200 randomly selected muscle fibers were measured under a 4x microscope equipped with a Dino-Eye eyepiece camera, and the data were analyzed with Dino Capture Version 2.0 software (AnMo Electronics Corporation, New Taipei City, Taiwan).

### 2.7. Ribonucleotide analysis

Muscle samples were homogenized in 0.6 M perchloric acid, and the homogenate was adjusted to pH 7.0 using KH₂PO₄ and 0.8 M KOH buffer [34]. The homogenate was centrifuged (10,000 × g) for 10 min at 4°C, and the resulting supernatant was analyzed for guanosine monophosphate (GMP), inosine monophosphate (IMP), hypoxanthine, and inosine with HPLC (Chromaster system, Hitachi, Tokyo, Japan) with a UV detector set at 210 nm. The separation was performed using a TSK Gel Amide 80 column (Tosoh, Tokyo, Japan) as the stationary phase, with a mobile phase consisting of 30:70 KH₂PO₄ to acetonitrile buffer. An external standard curve was used to quantify ribonucleotide content.

### 2.8. Free and total amino acid analysis

Free amino acids were measured following the methods outlined in AOAC [35] and ISO 13903 [36]. A 1 g muscle sample was homogenized in 25 mL of 70% ethanol and centrifuged at 10,000 × g for 20 min at 20°C. The resulting supernatant was evaporated, and 2 mL of borate buffer was added prior to derivatization. The sample was filtered with a syringe filter (0.45 µm) and injected into HPLC (LC-20A, Shimadzu, Japan) equipped with an RF-10A XL fluorescence detector and an Ultra-C18 column (5 µm, 250 × 4.6 mm, Restek, Bellefonte, PA, USA). The analysis was conducted at a flow rate of 1 mL/min, with the column oven maintained at 40°C. Fluorescence detection was performed at an excitation wavelength of 263 nm and an emission wavelength of 313 nm. Peak areas were quantified using a standard amino acid curve [37].

Total amino acids were determined according to the method described by Souphannavong et al. [38], with slight modifications. A 1 g meat sample was hydrolyzed with 30 mL of 6 M HCl containing phenol under reflux at 110–120°C for 24 h. The hydrolysate was filtered using Whatman No. 1 filter paper and concentrated to dryness using an evaporator. The residue was rinsed twice with 20 mL of water, evaporated to dryness, and dissolved in 20 mL of borate buffer. The pH was adjusted to 8.5 using 5 M NaOH. After derivatization, the sample was filtered with a syringe filter (0.45 µm) and analyzed using the same HPLC system and detection parameters as for free amino acids. Peak areas were quantified based on a standard amino acid curve.

### 2.9. Fatty acid analysis

Lipids were extracted using a 2:1 v/v chloroform-methanol mixture [39]. The fatty acids were transmethylated to fatty acid methyl esters (FAME) using 0.66 N KOH in methanol and 14% methanolic BF₃ [35]. Ethylnonadecanoate (SFA-013N, AccuStandard, New Haven, CT, USA) was used as an internal standard. FAME analysis was performed using a gas chromatograph (7890B, Agilent, Santa Clara, CA, USA) equipped with a fused silica capillary column (SPTM-2560, Supelco, Bellefonte, PA, USA; 100 m × 0.25 mm × 0.2 µm film thickness) and a flame ionization detector. The operating conditions were as follows: injector temperature, 240°C; detector temperature, 260°C; carrier gas, helium at a constant pressure of 110.32 kPa; split ratio, 10:1. The temperature program started at 60°C, increased to 170°C at 20°C/min, then to 220°C at 5°C/min, and finally to 240°C at 2°C/min [32]. FAME peaks were identified and quantified by comparing peak areas and retention times with those of known FAME standards (FAME Mix, C4-C24, Supelco, Bellefonte, PA, USA).

### 2.10. Thiobarbituric acid reactive substances (TBARS) determination

The TBARS assay was performed to determine lipid oxidation in meat samples stored at 4°C for 1, 3, and 5 days postmortem. A 10 g meat sample was homogenized with 35 mL of cold extraction solution containing 9% perchloric acid at 13,500 × g for 40 s. After homogenization, 25 mL of chilled water was added, and the mixture was homogenized for an additional 15 s. The solution was filtered via Whatman No. 2 filter paper. A 2 mL aliquot of the filtrate was homogenized with 2 mL of 0.02 M thiobarbituric acid (TBA) solution and heated at 80 ± 2°C for 60 min to form the malondialdehyde–TBA complex. The mixture was cooled under cold tap water for 10 min, and absorbance was measured at 530 nm with a UV-1280 UV-VIS spectrophotometer (Shimadzu, Kyoto, Japan). A blank sample containing 5 mL of distilled H_2_O and 5 mL of 0.02 M TBA solution was used for calibration. The malondialdehyde (MDA) concentration was quantified using a standard curve generated from tetraethoxypropane, and the results were expressed as mg MDA per kg of meat.

### 2.11. Statistical analysis

A power analysis was conducted prior to the trial. Based on the Altman’s normo-gram and G power analysis using the effect size, the minimum n, i.e., the sample size (replicate) per dietary group to detect meaningful changes at 95% confidence interval and 80% test power was 4. The actual test power was 95.6%. The trial followed a completely randomized design with five replicates per dietary group. Dietary treatment was treated as fixed effect while goats were treated as random effect. The normality of the data was evaluated using the Shapiro–Wilk test, and homogeneity of variance was assessed using Levene’s test; the results confirmed that the data were normally distributed and had homogeneous variances. Data were analyzed using PROC GLM of SAS (SAS Institute, Cary, NC, USA). The model is as follows:


$${\text{Y}}_{\text{ij}}=\;\mu +{\text{D}}_{\text{i}}+\epsilon_{\text{ij}}$$

Where

Y_*ij*_ = observed trait on the j^th^ goat receiving diet *i*

*μ* = overall mean

D_i_ = fixed effect of diet *i* (0, 2, 4%)

ε_*ij*_ = residual error

TBARS data were analyzed using the PROC MIXED procedure of SAS, with diet, storage time, and their interaction specified as fixed effects, and goat considered as a random effect. Storage time (days 1, 3, and 5) was treated as the repeated measure within each goat, since TBARS was assessed on the same muscle sample from each animal across different storage days. Accordingly, the statistical model in PROC MIXED was:


$${\text{Y}}_{\text{ijk}}=\mu +{\text{D}}_{\text{i}}+{\text{T}}_{\text{k}}+(\text{D}\times \text{T}{)}_{\text{ik}}+{\text{G}}_{\text{j}(\text{i})}+\epsilon_{\text{ijk}}$$

where

Y_ijk_ = TBARS value of goat *j* in diet *i* at storage time *k*,

μ = overall mean,

D_i_ = fixed effect of diet *i*,

T_k _= fixed effect of storage time *k*,

(D×T)_ik_ = diet × time interaction,

Gj(i) = random effect of goat *j* nested within diet *i*,

ε_ijk_ = residual error, modeled with an appropriate covariance structure to account for repeated measures.

Least square means were compared using the PDIFF option in SAS, with the level of significance set at *P* < 0.05.

## 3. Results

### 3.1. Carcass traits

There was no significant difference in the slaughter weight among the groups (Table 3), though HL-4 goats tended to have a higher slaughter weight than others (*P* = 0.074). The HL-4 goats had greater hot and cold carcass weights (*P* < 0.05), although dressing percentage did not differ significantly among the dietary treatments. Also, purge loss also showed no significant difference among treatments (*P* > 0.05). For carcass composition, there was a significant difference in shoulder percentage (*P* < 0.01), with HL-2 and HL-4 goats having higher values than HL-0 goats (Table 3).

**Table 3:** Carcass traits^1^ and physiochemical properties^1^ of *Longissimus* muscle in goats fed diets supplemented with hemp leaves.

Item	Level of hemp leaves (%)			RMSE	*p value*
	0	2	4		
Carcass traits^1^					
Initial weight (kg)	16.02	16.19	16.63	1.73	0.870
Slaughter weight (kg)	25.30	24.80	28.50	2.28	0.074
Hot carcass weight (kg)	11.80^b^	11.20^b^	13.75^a^	1.06	0.012
% hot carcass weight	46.85	45.18	48.18	2.35	0.202
Cold carcass weight (kg)	11.34^b^	10.82^b^	13.31^a^	1.06	0.013
% cold carcass weight	45.04	43.66	46.59	2.44	0.242
% purge loss	3.90	3.33	3.31	1.10	0.653
Loin (%)	10.24	8.97	11.38	1.60	0.123
Hide leg (%)	23.02	23.39	20.19	2.24	0.116
Chump (%)	8.39	8.38	7.98	1.55	0.912
Rack (%)	9.03	9.12	9.85	1.06	0.478
Shoulder (%)	5.76^b^	8.30^a^	7.15^a^	1.06	0.009
Fore leg (%)	21.10	20.25	20.03	1.47	0.518
Physiochemical properties^1^					
pH 45 min	6.17	6.06	6.34	0.22	0.196
pH_24_	5.94^ab^	5.71^b^	6.06^a^	0.19	0.044
Color					
L*	31.6	31.5	30.6	0.49	0.692
a*	9.3	8.9	8.6	0.32	0.751
b*	10.9	10.4	9.0	0.38	0.180
Drip loss (%)	4.38	4.29	4.27	0.30	0.989
Cooking loss (%)	36.2^a^	37.3^a^	31.9^b^	0.53	0.006
Shear force (N)^2^	51.32	62.49	45.24	12.36	0.067
Sarcomere length (µ)^2^	2.22	1.78	2.28	0.27	0.115
Muscle fiber diameter (µ)	64.1	65.8	70.8	8.08	0.473
Chemical composition (%)					
Dry matter	24.22	25.57	25.02	0.42	0.443
Crude protein	21.50	20.04	20.99	0.42	0.381
Ether extract	2.44	3.24	3.51	0.43	0.614
Ash	1.08	1.09	1.10	0.01	0.829

a,b LSmeans in a row with different superscripts differ significantly (*P* < 0.05). RMSE, root mean square error. ^1^Each value is a least square mean of five replicates. ^2^ Shear force and sarcomere length data were analyzed using pH_24_ as a covariate, with a mean pH value of 5.89.

### 3.2. Physicochemical properties

The physicochemical properties of LTL muscles from goats supplemented with graded amounts of hemp leaves are shown in Table 3. The addition of hemp leaves had no effect on the color, drip loss, chemical composition, shear force, sarcomere length or muscle fiber diameter of the goats (*P* > 0.05). The HL-4 meat had a significantly higher ultimate pH (pH 24 h post-mortem) compared to the HL-2 meat, while the pH of the control meat did not differ from that of the other groups. The HL-4 meat had lower cooking loss than the other groups.

### 3.3. Ribonucleotides

Ribonucleotide levels in LTL muscle of goats fed graded levels of hemp leaves are shown in Table 4. No significant differences in the concentrations of major ribonucleotides, including IMP, GMP, inosine and hypoxanthine were found between dietary groups.

**Table 4:** Ribonucleotides^1^ (mg/100 g) in *Longissimus muscle* of goats fed diets supplemented with hemp leaves.

Item	Level of hemp leaves (%)			RMSE	*p value*
	0	2	4		
Hypoxanthine	11.34	13.34	12.15	3.91	0.707
Inosine	118.6	129.1	120.1	23.27	0.754
Inosine monophosphate	380.2	422.3	370.3	72.86	0.529
Guanosine monophosphate	0.56	0.79	0.99	0.47	0.411

RMSE, root mean square error. ^1^Each value is a least square mean of five replicates.

### 3.4. Free and Total amino acids

The free and total amino acid profiles of LTL muscle from goats supplemented with graded amounts of hemp leaf are shown in Tables 5. Hemp leaf supplementation had no significant effect on the concentrations of free amino acids or total amino acids in LTL muscle.

**Table 5:** Free and total amino acids^1^ (mg/g) in *Longissimus muscle* of goats fed diets supplemented with hemp leaves.

Item	Level of hemp leaves (%)			RMSE	*p value*
	0	2	4		
Free amino acid					
Aspartic	0.11	0.10	0.12	0.03	0.693
Glutamic	1.29	1.11	1.54	0.44	0.381
Histidine	0.49	0.41	0.50	0.17	0.706
Serine	0.15	0.13	0.16	0.05	0.678
Arginine	0.59	0.49	0.66	0.23	0.548
Glycine	0.11	0.09	0.12	0.04	0.633
Threonine	0.11	0.09	0.12	0.04	0.737
Alanine	0.35	0.27	0.41	0.15	0.407
Proline	0.27	0.26	0.28	0.02	0.756
Tryptophane	0.96	0.89	1.29	0.54	0.527
Lysine	0.11	0.35	0.12	0.35	0.491
Valine	0.12	0.12	0.18	0.08	0.542
Methionine	0.22	0.23	0.27	0.08	0.639
Isoleucine	0.17	0.12	0.24	0.13	0.471
Leucine	0.46	0.38	0.52	0.23	0.697
Phenylalanine	0.98	0.61	0.96	0.38	0.262
EAA^2^	3.62	3.22	4.18	1.56	0.665
NAA^2^	2.88	2.45	3.28	0.79	0.335
Umami	1.41	1.21	1.65	0.44	0.365
Sweet	0.98	0.85	1.08	0.29	0.516
bitter	4.11	3.61	4.73	1.75	0.646
Total amino acid					
Aspartic	1.96	1.67	1.99	0.62	0.695
Glutamic	3.67	2.46	4.72	1.47	0.113
Histidine	6.93	6.62	8.46	2.36	0.497
Serine	1.68	1.84	1.98	0.60	0.772
Arginine	3.81	3.34	5.72	1.82	0.173
Glycine	2.86	2.63	3.44	1.03	0.516
Threonine	1.61	1.78	2.01	0.67	0.687
Alanine	1.57	1.32	2.41	1.16	0.384
Proline	8.15	7.42	9.85	1.98	0.225
Lysine	1.29	1.36	1.70	0.48	0.432
Valine	1.66	1.74	2.50	0.98	0.412
Methionine	2.26	2.53	2.81	0.53	0.343
Leucine	7.26	7.28	9.94	3.55	0.469
Phenylalanine	2.99	2.89	3.40	0.82	0.647
EAA^3^	5.10	5.02	6.14	1.57	0.453
NAA^3^	5.68	4.28	7.26	1.91	0.216

^1^Each value is a least square mean of five replicates. EAA^2^ = histidine + tryptophane + lysine + valine + methionine + isoleucine + leucine + phenylalanine + threonine, NAA^2^ = aspartic + glutamic + serine + arginine + glycine + alanine + proline, umami = aspartate + glutamate, sweet = alanine + glycine+ proline + threonine + serine, bitter = arginine + histidine + isoleucine + leucine + lysine + methionine + phenylalanine + tryptophane + valine. RMSE, root mean square error. ^1^Each value is a mean of five replicates. EAA^3^ = histidine + lysine + valine + methionine + leucine + phenylalanine + threonine, NAA^3^ = aspartic + glutamic + serine + arginine + glycine + alanine + proline, RMSE, root mean square error.

### 3.5. Fatty acid composition

The major saturated fatty acids (SFAs), monounsaturated fatty acids (MUFAs), and polyunsaturated fatty acids (PUFAs) remained stable across dietary treatments (P > 0.05; Table 6). The HL-2 meat had a higher concentration of C20:2n-6 compared with HL-0 and HL-4 meats (Table 6). The concentration of C22:0 was lower (P < 0.05) in HL-0 meat compared with HL-2 meat, while the HL-4 meat showed no significant difference from either HL-0 or HL-2 meats. In addition, the concentration of C20:4n-6 was lower (P < 0.05) in HL-0 meat than in HL-2 and HL-4 meats. Overall, HL-2 meat exhibited higher total SFA and PUFA contents compared with HL-0 and HL-4 meats.

**Table 6:** Fatty acids composition of *Longissimus* muscle of goats fed diets supplemented with hemp leaves^1^.

Item (mg/100 g wet sample)	Level of hemp leaves (%)			RMSE	*p value*
	0	2	4		
c14:0	46.88	53.06	48.31	6.14	0.739
c14:1	15.93	19.10	12.33	2.35	0.32
c15:0	9.97	15.42	12.80	1.80	0.292
c16:0	305.20	380.59	297.41	39.54	0.473
c16:1	91.44	112.71	91.76	12.13	0.553
c17:0	42.90	66.70	47.31	7.19	0.237
c17:1	28.76	42.92	31.85	5.14	0.339
c18:0	358.59	499.83	377.17	49.92	0.321
c18:1n9c	922.10	1251.66	897.79	140.11	0.800
c18:2n6c	51.90	64.80	42.48	8.79	0.428
c20:0	1.18	1.50	1.11	0.16	0.396
c18:3n6	0.41	0.66	2.51	0.83	0.135
c20:1n9	3.22	2.51	3.05	0.45	0.597
c18:3n3	4.46	6.90	5.33	5.27	0.323
c21:0	19.55	20.69	18.94	2.89	0.084
c20:2n-6	5.23^b^	8.60 ^a^	5.89 ^b^	0.93	0.030
c22:0	2.13^b^	5.48^a^	3.75^ab^	1.59	0.044
c20:3n6	2.74	3.72	2.21	0.55	0.600
c22:1n9	0.11	0.09	0.00	0.04	0.387
c20:4n6	17.19^b^	39.00^a^	30.69^a^	3.96	0.045
c23:0	0.16	0.00	0.00	0.05	0.281
c22:2	0.00	0.05	0.00	0.02	0.281
c20:5n3	2.05	3.41	1.77	0.55	0.230
c22:6n3	0.98	1.37	1.03	0.38	0.839
MUFA	1061.56	1428.99	1036.78	124.7	0.190
PUFA	84.96^b^	128.51^a^	91.91^b^	20.60	0.039
SFA	786.40^b^	1043.27^a^	806.80^b^	136.21	0.038
PUFA:SFA	0.11	0.12	0.11	0.02	0.242
n3	7.49	11.69	8.13	1.69	0.303
n6	72.24^b^	108.18^a^	77.89^b^	1.74	0.040
n6:n3	9.64	9.26	9.58	1.4	0.787

a,b Lsmeans with different superscripts in a row are significantly different (*P* < 0.05). RMSE, root mean square error. ^1^Each value is a least square mean of five replicates.

### 3.6. Lipid oxidation

**Fig 1:**
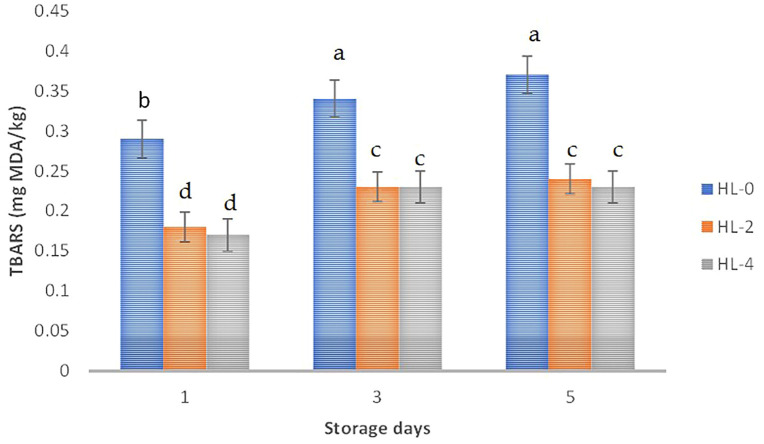
Lipid oxidation during chill storage of *Longissimus* muscle of goats fed diets supplemented with hemp leaves. Standard error bars having different superscripts are significantly different (*p* < 0.05). HL-0, goats without HL supplementation; HL-2, goats supplemented with 2% HL in concentrate; HL-4, goats supplemented 4% HL in concentrate.

On days 1, 3 and 5, the LTL muscles from goats supplemented with 2% and 4% hemp leaves had lower TBARS levels than the control LTL (*P* < 0.01; Fig 1). Regardless of the treatment, the TBARS value on days 3 and 5 did not differ but was higher than that of day 1 postmortem. The treatment-storage interaction was not significant.

## 4. Discussion

The slaughter weight of goats was not affected by dietary supplementation with hemp leaves, consistent with comparable feed intake, nutrient digestibility, and overall growth performance among treatments [40]. However, goats fed 4% hemp leaves exhibited greater hot and cold carcass weights than those fed 0 or 2% hemp leaves, suggesting shifts in nutrient partitioning rather than overall body growth. Hemp leaves are rich in polyphenols and flavonoids [8,9] that enhance antioxidant status [10,11,22] and reduce systemic oxidative and metabolic stress [41], thereby sparing nutrients for lean tissue deposition and promoting muscle protein accretion. Moreover, cannabinoids and the endocannabinoid system (ECS) regulate energy balance and nutrient partitioning in ruminants via CB1 and related signaling pathways [42,43]. Transcriptomic studies indicate that dietary cannabinoids from spent hemp biomass can alter hepatic gene expression related to lipid metabolism and ECS signaling, supporting potential effects on tissue lipid handling [44]. Activation of CB1 has been associated with enhanced adipogenesis and reduced lipolysis, while modulation of ECS tone by phytocannabinoids may optimize lipid turnover and hepatic lipid synthesis [45,46]. These mechanisms may favor lean tissue accretion and reduce non-carcass components (e.g., gut fill, visceral fat), thereby increasing the proportion of slaughter weight represented by carcass weight. The increased shoulder proportion observed in HL-2 and HL-4 goats suggests that hemp leaf supplementation may influence the regional distribution of muscle mass, potentially through effects on muscle fiber development.

Consistent results have been reported with other herbal supplements. Supplementation with *Andrographis panniculata* leaves and stems improved hot and cold carcass weights in goats [47], while *Rosmarinus officinalis* leaves enhanced cold carcass weights in lambs [48]. In contrast, hemp stubble [12] and spent hemp biomass [15] had no significant effects on carcass characteristics in lambs. Hempseed meal also showed variable effects on carcass traits in goats, with a study reporting no effect [13] and another reporting reduced carcass weight [14].

Dietary hemp leaf supplementation had no effect on meat color, drip loss, chemical composition, or muscle fiber diameter in goats. This may be attributed to the similar energy and protein content of the dietary treatments, as well as the comparable growth rates, and uniform husbandry and slaughter conditions [40]. In line with our findings, the supplementation of *Andrographis paniculata* [49], polyphenol [50] and green tea by-products [51] did not affect drip loss or chemical composition in goats. Similarly, *Rosmarinus officinalis* leaf supplementation did not influence drip loss in lambs [48]. However, supplementation with spent hemp biomass at 10% had no effect on meat quality, whereas inclusion at 20% increased cook loss in lambs [15].

The HL-4 meat had a significantly higher ultimate pH compared to the HL-2 meat, while the pH of the control meat did not differ from that of the other groups. This suggests that the 2% and 4% hemp leaf supplementation exert contrasting effects on the ultimate pH in goats. The higher muscle pH observed in HL-4 meat may be attributed to its elevated polyphenol content, which protects proteins from oxidative damage, thereby preserving post-mortem muscle cell integrity and maintaining enzyme activity, preventing excessive pH decline in the meat. Also, polyphenols can interact with muscle proteins by binding or cross-linking [52], which may influence protein degradation and water-holding capacity. These interactions could indirectly affect the rate of post-mortem acidification, contributing to variations in meat pH. The higher pH may explain the lower cooking loss in the HL-4 meat. When meat has a higher pH (above 5.8), the proteins carry a net charge, which allows them to hold more water within the muscle fibers [53,54]. This improves the meat’s water-holding capacity, resulting in lower cooking loss. The lack of changes in sarcomere length, shear force and color values despite differences in pH suggests that the influence of pH on muscle properties was likely overridden or balanced by other physiological or biochemical factors. Similar to our findings, spent hemp biomass supplementation did not alter the meat shear force and color of lambs [15]. In addition, supplementation of *Mitragyna speciosa* (Korth) havil leaves did not affect the color values and shear force in goats [55].

Ribonucleotide levels in the LTL muscle of goats fed graded amounts of hemp leaves showed no significant differences. Ribonucleotides are products of muscle metabolism that are influenced by genetic factors, muscle fiber type and metabolic activity in muscle tissue [34,56]. Since ribonucleotides are synthesized endogenously during muscle energy metabolism [57], a dietary supplement, such as hemp leaf, may not significantly alter their content unless it contains components that directly affect energy metabolic pathways or nucleotide metabolism. While hemp leaves are rich in bioactive compounds such as cannabinoids, and flavonoids [8], these compounds may not directly affect metabolic processes involved in ribonucleotide production in muscle tissue. For a dietary supplement to influence nucleotide content, it would need to provide specific precursors or significantly alter nucleotide turnover processes, which may not be achieved with hemp leaf at 4% supplementation. This result is consistent with the similar pH_45_ levels observed in LTL muscle across treatments and is in line with previous research showing no effect of diet on ribonucleotide concentrations in beef [32].

Free amino acids are of crucial importance for the flavor profile of meat, as they directly influence the taste and indirectly contribute to the formation of flavor compounds during cooking [53,54,58,59]. In this study, supplementation with hemp leaf had no significant effect on free or total amino acid content in LTL muscle of goats. This observation could be due to the similar protein contents of the diets as well as the comparable protein digestibility [40], protein metabolism in the rumen and the specific metabolic pathways that regulate the uptake of amino acids and their incorporation into muscle tissue. Similar results were observed in previous studies in which the supplementation with *Broussonetia papyrifera* leaves did not alter the amino acid profile of beef [60].

The fatty acid profile of ruminant meat is primarily determined by ruminal biohydrogenation, endogenous fatty acid synthesis from volatile fatty acids produced during rumen fermentation, and *de novo* fatty acid synthesis in adipose tissue [61–63]. The stability of the major SFA, MUFA, and PUFA across dietary treatments suggests that the inclusion of hemp leaves up to 4% did not markedly alter ruminal biohydrogenation, which contributes approximately 60–90% of the fatty acids available for tissue deposition [61]. Goats fed 2% hemp leaves exhibited higher concentrations of C20:2n-6 compared with those fed 0% and 4% hemp leaves. This likely reflects enhanced elongation and desaturation of C18:2n-6 via hepatic enzymatic activity, primarily involving elongase and desaturase enzymes that catalyze the sequential conversion of C18:2n-6 to C20:2n-6 and its downstream metabolites [64–66]. Moderate supplementation likely provided an optimal substrate supply and sufficient micronutrients, such as zinc and magnesium, which serve as cofactors for Δ6-desaturase and elongase enzymes [67]. At higher inclusion level (4%), potential anti-nutritional factors may have impaired lipid metabolism, reducing enzyme efficiency.

Similarly, the higher concentration of C22:0 in HL-2 meat compared with HL-0 may indicate stimulation of fatty acid elongation pathways. Phytochemicals and phytosterols in hemp leaves can activate peroxisome proliferator-activated receptors (PPARs), enhancing hepatic lipid oxidation and elongation [68,69]. The absence of further increases at 4% inclusion may reflect inhibitory effects of excessive polyphenols.

Elevated C20:4n-6 levels in HL-2 and HL-4 meat compared with HL-0 suggest increased conversion of C18:2n-6 to its long-chain derivative via Δ5- and Δ6-desaturase activity [64–66]. Dietary hemp seeds have been shown to modify milk fatty-acid composition and indices of desaturase/elongase activity [70]. In addition, hemp-derived flavonoids and polyphenols may protect PUFA from oxidative degradation, consistent with the lower TBARS values observed in HL-supplemented goats. This antioxidant protection likely contributed to higher deposition of C20:4n-6 in muscle tissues.

The higher total SFA and PUFA contents observed in HL-2 meat compared with the HL-0 and HL-4 could be attributed to the elevated concentrations of individual fatty acids within these classes. This pattern suggests that moderate hemp leaf supplementation enhanced lipid metabolic efficiency, supporting balanced elongation and desaturation of fatty acid substrates. These results agree with previous studies in which supplementation with medicinal plants altered muscle fatty acids in goats [49,50,55] and sheep [48,71].

TBARS, a measure of malondialdehyde formed during lipid oxidation, is widely used to assess oxidative stability [72]. Hemp leaf supplementation reduced TBARS values during 5 days of chilled storage, indicating lower lipid oxidation in chevon, likely due to the antioxidant activity of hemp flavonoids and polyphenols [8,9]. By delaying oxidation, hemp may help preserve quality, flavor, and shelf life, consistent with findings from *Andrographis paniculata* [49] and polyphenols [50]. TBARS values increased from day 1 to days 3 and 5, reflecting the depletion of antioxidant defenses and the accumulation of pro-oxidants (e.g., iron from myoglobin), which accelerate lipid oxidation and yield secondary products like malondialdehyde [73].

Although the Δ⁹-THC concentrations in the goat diets used in the present study were extremely low (0.00036–0.00055%), they were well below the recommended threshold of 0.001% for livestock feed [23,24]. However, evidence indicates that even minimal dietary inclusion of hemp by-products (<0.01%) can result in detectable accumulation of Δ⁹-THC in animal-derived products such as meat [12,74,75] and milk [76–78]. Therefore, while the levels used in this study are unlikely to pose immediate food safety concerns, the potential transfer of Δ⁹-THC to goat meat cannot be completely excluded. This represents a limitation of the current study and warrants further investigation. Another limitation lies in the analytical approach: although HPLC-UV effectively quantified major cannabinoids such as cannabidiolic acid, cannabidiol, cannabinol, Δ⁹-tetrahydrocannabinol, and Δ⁹-tetrahydrocannabinolic acid A, it is less sensitive than LC-MS/MS. Consequently, the presence of other minor cannabinoids in hemp leaves cannot be ruled out, as their concentrations may have been below the detection threshold of the method used.

## 5. Conclusions

Feeding 2% or 4% hemp leaves had different effects on the carcass characteristics and meat quality of goats. Goats fed 4% hemp leaves had significantly heavier hot and cold carcasses. The dietary treatments had no effect on color, chemical composition, muscle fiber diameter, drip loss, ribonucleotide content, or free and total amino acid profile. HL supplementation improved C20:4n-6 while the H-2 meat had higher total SFA and PUFA compared to other meats. However, the HL-4 meat had a higher ultimate pH and lower cooking losses. In addition, hemp leaf supplementation reduced TBARS levels, indicating improved oxidative stability. Overall, both the 2% and 4% hemp leaf supplementation had a positive effect on certain carcass characteristics and meat quality, particularly in terms of fatty acids, oxidative stability and moisture retention. Hemp leaves can be included at 2–4% of goats’ diets to improve carcass traits, oxidative stability, and certain aspects of meat quality. Further studies are needed to determine the concentration of Δ9-THC in goat meat to ensure consumer safety before large-scale adoption.
